# Does the “Root Removal First” strategy prevent postoperative complications in the surgical removal of impacted mandibular third molars in the Pell and Gregory class C and horizontal position? — a randomized clinical trial

**DOI:** 10.1186/s12903-023-03086-9

**Published:** 2023-06-14

**Authors:** Bing Wang, Rui Sun, Tingting Li, Yuqi Sun, Linwei Zheng, Jihong Zhao

**Affiliations:** 1 The State Key Laboratory Breeding Base of Basic Science of Stomatology (Hubei-MOST) & Key Laboratory of Oral Biomedicine Ministry of Education, School & Hospital of Stomatology, Wuhan University, 237 Luoyu Road, Wuhan, 430079 China; 2 Department of Oral Surgery, School & Hospital of Stomatology, Wuhan University, Wuhan, China

**Keywords:** Impacted mandibular third molar, Extraction, Mandibular second molar, Alveolar bone, Inferior alveolar nerve

## Abstract

**Objective:**

To evaluate the clinical outcomes of the “Root Removal First” strategy in the surgical removal of impacted mandibular third molar (IMTM) in the class C and horizontal position.

**Materials and methods:**

A total of 274 cases were finally included in the statistics. The positions of IMTM in the horizontal position were confirmed by cone-beam computed tomography (CBCT). Cases were randomly divided into two groups: the “Root Removal First” strategy was applied in the new method (NM) group, and the conventional “Crown Removal First” strategy was executed in the traditional method (TM) group. The clinical information and relevant data upon follow-up were recorded.

**Results:**

The duration of the surgical removal and the incidence rates of lower lip paresthesia in the NM group were significantly lower than those in the TM group. The degree of mobility of the adjacent mandibular second molar (M2) in the NM group was significantly lower than that in the TM group at 30 days and 3 months post-operation. The distal and buccal probing depth of the M2, as well as the exposed root length of M2 in the NM group, were significantly lower than those in the TM group 3 months post-operation.

**Conclusions:**

The “Root Removal First” strategy can reduce the incidence rate of inferior alveolar nerve injury and periodontal complications of the M2 in the surgical removal of IMTM in class C and horizontal position with high efficiency.

**Trial registration:**

ChiCTR2000040063.

## Introduction

Third molars (M3s) account for about 98% of all impacted teeth. The evolutionary question is whether human jaw size has decreased below a threshold such that our M3s lack space to develop [1]. Therefore, the prevalence of impacted M3, which is the last tooth to erupt is increasing, especially in the mandibular third molars [2]. Pathologies that are associated with IMTMs included pericoronitis, space infection, osteomyelitis of the jaw, crowding of the dentition, cyst. More frequently, the impaction may contribute to dental caries of the adjacent second molars (12.6%), the distal deep periodontal pocket (8.9%), distal bone loss (9.7%), external root resorption of mandibular second molars (20.17%-52.9%) [3, 4]. At present, surgical removal of the IMTM is the conventional and preferred treatment to solve the aforementioned clinical problems caused by IMTM [5]. However, surgical removal of the IMTM can be complex, particularly in deeply impacted cases, which may often lead to undesirable complications. Above all, IMTMs in class C (based on Pell-Gregory classification) and horizontal position (or deeply IMTM) are relatively more difficult and are often associated with various complications, especially inferior alveolar nerve injury (3.6–8.0%) [6], and periodontal problems of the adjacent mandibular second molar (M2). However, the periodontal problems of M2 following the surgical removal of the IMTM are usually overlooked by the surgeon. It was reported that the incidence of distal bone defect of M2 exceeding 4 mm after IMTM removal was 32.1%, the incidence of distal deep periodontal pocket was 43.3% [7], and the average increase in probing depth was 5.4 ± 1.9 mm [8].

In order to promote better periodontal healing of the distal aspect of the M2, the osteotomy should not be too close to the M2. Care should be taken to avoid iatrogenic injuries to the alveolar crest of the adjacent M2. In this context, the surgical exposure of the root of the IMTM may be better than the crown. Therefore, the removal of the roots would be relatively easier as the resistance has been eliminated, and the friction between the roots and the inferior alveolar nerve may be reduced indirectly. Hence, we named it, explicitly, the “Root Removal First” Strategy. To investigate the clinical outcomes of this strategy, we compared this innovative method to the traditional “Crown Removal First” strategy. And it was shown to reduce the complications such as inferior alveolar nerve injury and extraction-related periodontal problems of M2.

## Materials and methods

### Subjects

This randomized controlled trial was conducted from January 2021 to June 2022 in the Department of Oral Surgery, Hospital of Stomatology, Wuhan University. The study followed the Declaration of Helsinki on medical protocol and ethics, and the study was approved by the Regional Ethical Review Board of the Ethics Committee of the Hospital of Stomatology, Wuhan University (No. 2020B72). The trial was also registered on the Chinese Clinical Trials Registry on 19/11/2020, with the registration number: 2000040063.

The trial design is parallel, and the allocation ratio is 1:1. There were no significant changes to both methods after the trial commencement.

### Eligibility criteria for participants

**Fig. 1:**
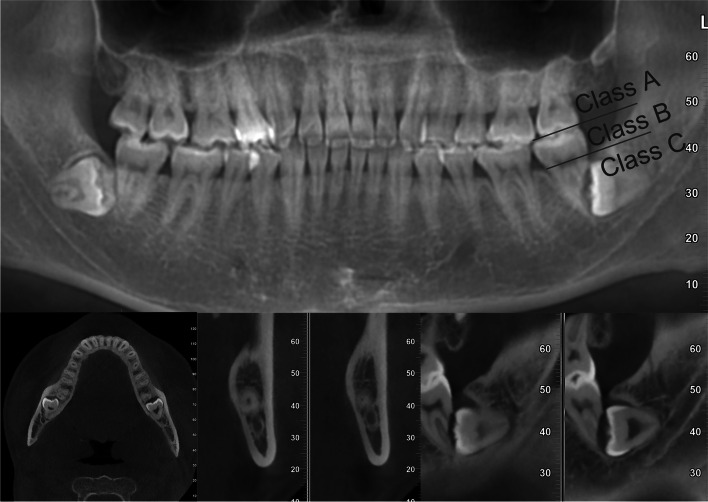
Preoperative CBCT image. #38 was in a horizontal position and the crowns were located below the cervical line of #37

• The inclusion criteria were as follows:

- 1) Patients are indicated for the surgical removal of IMTMs and agreeable to sign an informed consent.
- 2) Patients aged between 18 to 65 years old.
- 3) CBCT demonstrated that the IMTM was in horizontal position and the crown was located below the cervical line of the M2s (Class C) (Fig 1).
- 4) There was no acute soft tissue and periodontal inflammation of the posterior molars, and the M2 was firm.

• Patients were excluded in the following cases:

- 1) Patients were not willing to be enrolled in the study.
- 2) Patients with contraindications for tooth extraction.
- 3) Patients did not attend the post-operative follow-up.

The data were collected in the Department of Oral Surgery, Hospital of Stomatology, Wuhan University.

The new method (NM) group is the “Root Removal First” strategy and the traditional method (TM) group is the “Crown Removal First” strategy. Patients were assigned to each group using a computer-generated randomization list (a computer-generated block randomization technique). An independent clinician generated a random allocation sequence and all participants were kept blinded. The allocation sequence was concealed in sequentially numbered, opaque, and sealed envelopes, which were kept by the research officer. The sealed envelope containing the allocation sequence was opened by the clinician once the informed consent for this research was obtained. Both groups were operated by a single operator.

### Modes of anesthesia

Each patient was operated under inhalation sedation with nitrous oxide, and local infiltration anesthesia was administered to the buccal, lingual, and retromolar areas with by 4% Articaine (1:100,000 adrenaline) (Primacaine TM, France).

### The “Root Removal First” strategy was applied in the NM group

**Fig. 2:**
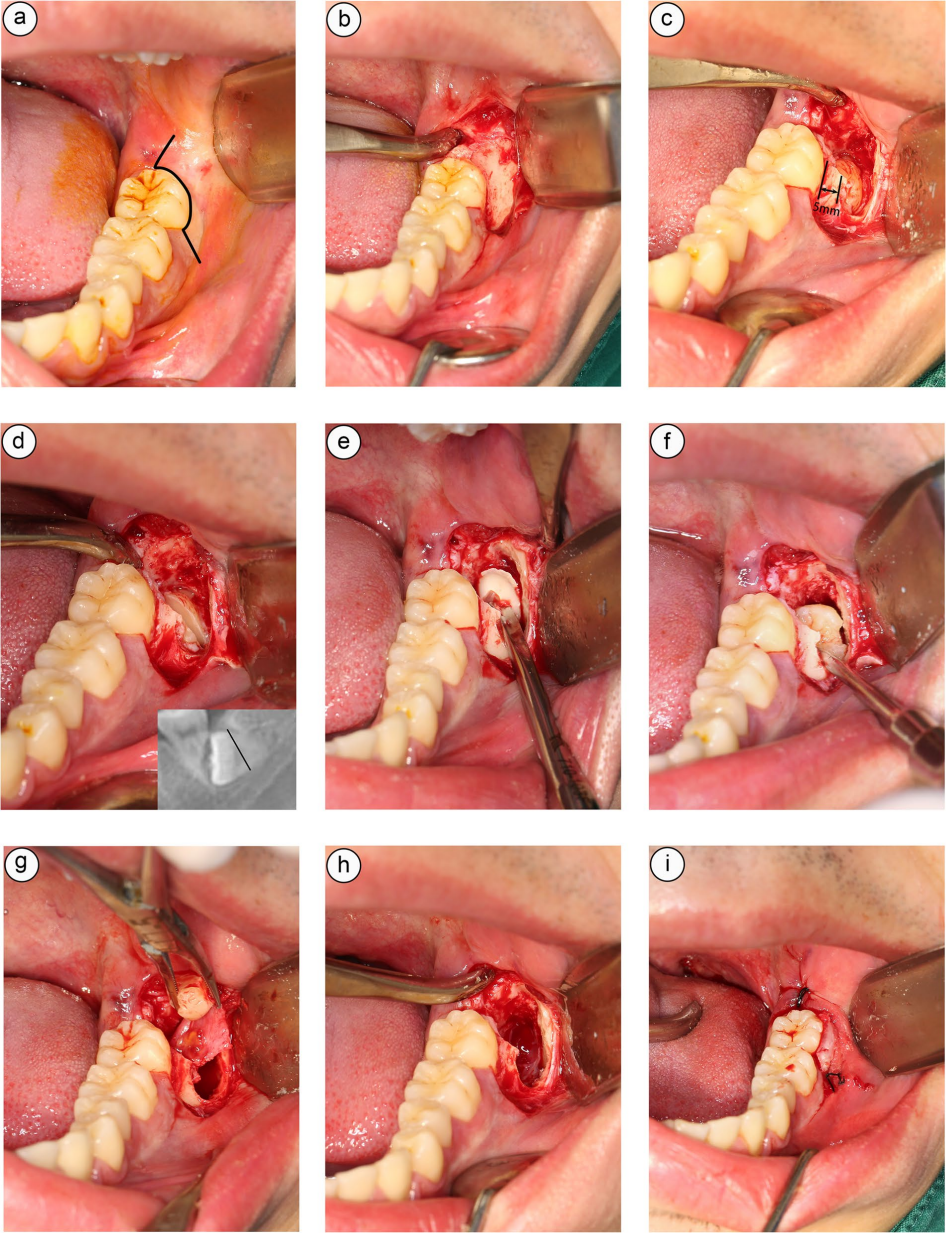
The extraction of 38 according to the “Root removal first” strategy. **a** Preoperative incision was designed. **b** Exposed the bone surface of the IMTM. **c** Bone window creation. **d** Impacted tooth separation. **e** Root removal. **f** Crown removal. **g** Dental follicle removal. **h** Irrigated the alveolar socket. **i** Sutured the wound

① Incision design: A longitudinal gingival incision was made from the mesio-buccal side of the M2, and an angular incision was made along the gingival margin to the retromolar pad. The mucoperiosteal flap was then raised buccally, and the buccal and occlusal aspects of the alveolar bone of the IMTM was exposed (Fig. 2a, b).

② Bone window creation: A bony window was then created to expose the IMTM by piezosurgery. In order to preserve at least 5 mm height of the buccal alveolar crest of M2, the main part of the bony window could only be made in the distal part of M2 using piezosurgery. The cervical region and part of the roots of IMTM would then be automatically exposed. Nevertheless, the main part of the crown was yet to be revealed. Subsequently, an extended micro bony window was made buccally to expose part of the crown of IMTM. (Fig. 2c).

③ Impacted tooth separation: The buccal and lingual bulbosities of the crown were both exposed. Similar to the tooth preparation for prosthetic crown, the circumference of the root was reduced by the fissure bur. IMTM was sectioned from the root with a fissure bur from the top to the bottom. The cutting line was at 45°-60° with the long axis of the tooth and the groove’s width was about 3 mm (Fig. 2d).

④ Root removal: The elevator was placed in the created gutter and the roots were elevated upward (Fig. 2e). The roots were further sectioned into buccal and lingual parts, if the roots remains firm during elevation. The main principle of this method is to reduce root resistance as much as possible. The roots may be fractured during the elevation. However, the crown could be luxated distally following removal of the roots.

⑤ Crown removal: After the root was removed, an elevator was placed in the created groove of the mesio-buccal micro bony window. The crown was luxated distally by rotating the elevator. If necessary, the crown can be sectioned again, and the segments can be removed in pieces (Fig. 2f).

⑥ Wound care: The residual dental follicle and debris was removed completely(Fig. 2g). The residual alveolar bone or bony spicules was smoothened, the area was irrigated with normal saline (Fig. 2h) and sutures are placed to achieve primary closure (Fig. 2i).

### The conventional “Crown Removal First” strategy was applied in the TM group

In TM group, as the crown was removed prior to the root(s), the incision and the creation of a bony window should be designed accordingly to allow sufficient exposure of the the crown of the IMTM. The wound care was the same as we have described in the NM group.

Standard postoperative care and instructions were given to both groups of patients routinely. Patients were instructed to remove the sutures at the second follow-up appointment (day 7).

### Observation and evaluation

Relevant dental and medical history of the patients, the positions, diagnosis, and radiographic examination of teeth were recorded before the treatment. The relationship between IMTM and the mandibular canal was also recorded [9]. The operation time was documented from the incision to the end of the wound closure. The sequence of the root or crown removal was also recorded.

The patients followed a standard postoperative review protocol, and incidence of postoperative hemorrhage [10], trismus [11], infection [12], and alveolar osteitis [13] were documented (if any). The patients were also required to rate the pain at 24 h and 48 h postoperatively using a visual analog scale (VAS), which ranges from 0 (no pain) to 10 (highest unbearable pain) [14]. Injury of the inferior alveolar nerve was evaluated according to the modified British Medical Research Council Neurological Dysfunction criteria at day 7 post-operatively [15]. The degree of the mobiligy of M2 were assessed at 30 days and 3 months post-operatively [16]. The distal and buccal probing depth [17], and the length of root exposure of the M2 were recorded at 3 months post-operation [18]. No changes to the trial outcomes were observed after the trial commencement.

### Statistical analysis

The student t-test was used for the comparison of measurement data by SPSS 26.0 (IBM, New York, NY). The Chi-square test was used for the comparison of enumeration data, and Fisher’s exact test was used for the comparison of enumeration data if the expected frequency of 25% or more of the cells is lower than 5., The rank-sum test was used for the comparison of grade data. A *P*-value of < 0.05 was considered statistically.

### Sample size/power calculation

The sample size was estimated considering a test power of 80%, a confidence interval of 95%, and an error of 5%, based on the ratio of inferior lip numbness rate in our previous pilot study (*n* = 38 patients). The Inferior lip numbness rate was 1/19(0.053) in the NM group and 3/19 (0.158) in the TM group.

For the determination of the sample size, the following formula was used:

$$n=\frac{{[{Z}_{\frac{\alpha }{2}}\sqrt{2\overline{p }\left(1-\overline{p }\right)}+{Z}_{\beta }\sqrt{{p}_{1}\left(1-{p}_{1}\right)+{p}_{2}(1-{p}_{2})}}^{2}}{{({p}_{1}-{p}_{2})}^{2}}$$

Z_0.025_≈1.96, Z_0.8_≈0.84, p_1_ = 0.03, p_2_ = 0.13, as the case number in both groups are the same, the sample size number is 132 in each group, the dropout rate is assumed as 10%. The dropout-inflated enrollment sample size is 147 for each group.

### Interim analyses and stopping guidelines

Statically analysis was performed after every 30 completed cases. If the NM group showed a worse outcome, the trial would be discontinued.

## Results

**Fig. 3:**
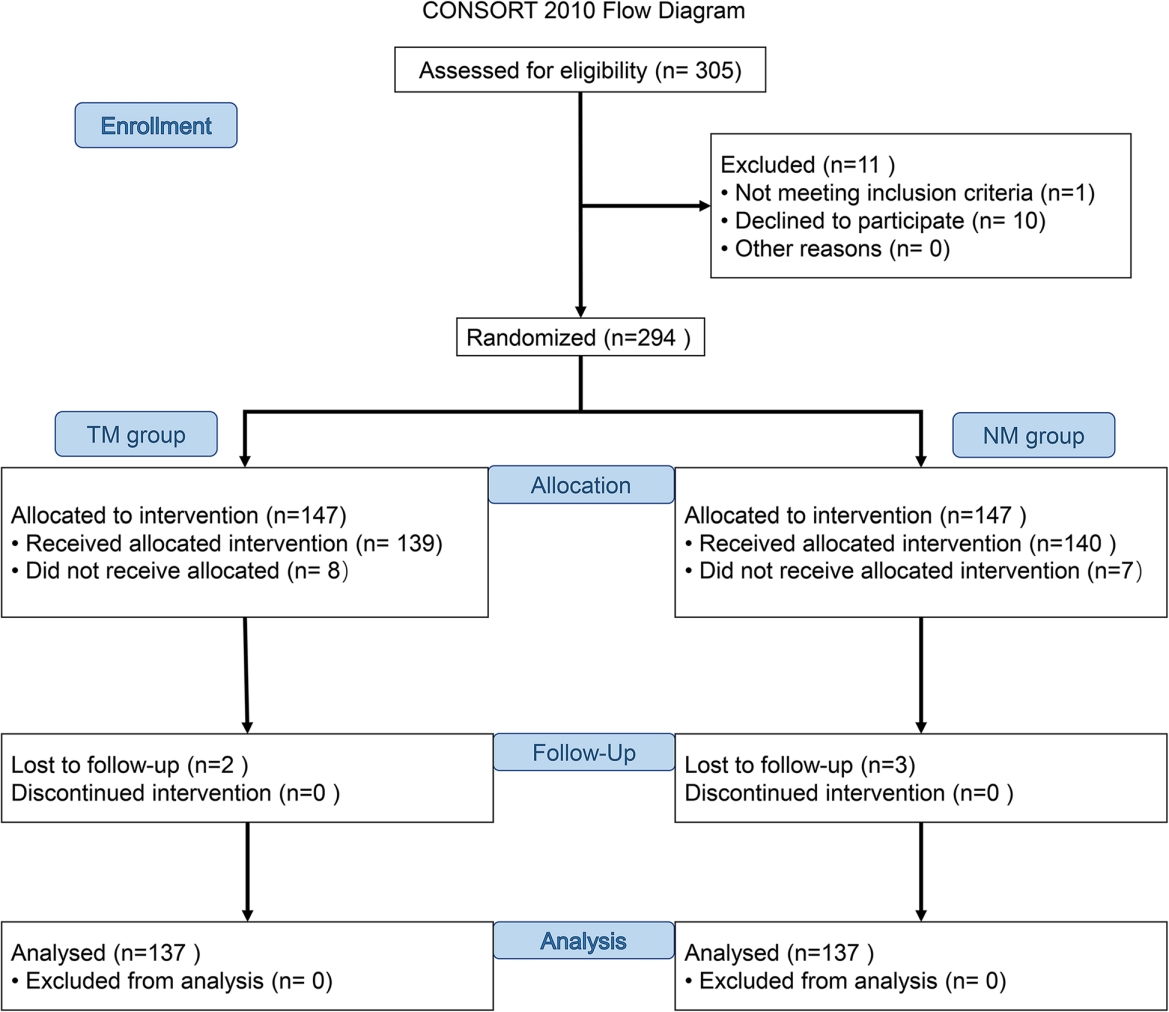
The CONSORT flowchart

During the enrollment, a total of 305 patients were assessed for eligibility. 1 patient did not meet inclusion criteria because of the presence of dentigerous cyst. 10 patients refused to participate. 294 cases of IMTMs in class C and horizontal position were randomly divided into the NM group and TM group. 8 in TM group and 7 in NM group was excluded as the patients were anxious about the post-operative pain and the potential nerve injuries. 2 in TM group and 3 in NM group patients failed to attend the follow-up after operation. A total of 274 cases were finally included in the statistics (Fig. 3). The 137 patients in the NM group included 65 males and 72 females, aged from 19 to 48 years, with an average of 28.3 years. The 137 patients in the TM group included 63 males and 74 females, aged from 18 to 55 years, with an average of 29.7 years. The relationship between mandibular canal and IMTM, the cortical bone status of the mandibular canal in the apical region of IMTM on CBCT, and the probing depth were also shown in Table 1. The trial ended after the enrollment of 294 cases.

**Table 1:** Basic clinical characteristics of 274 patients

	**Total (*n* = 274)**	**NM group (*n* = 137)**	**TM group (*n* = 137)**
**Age**			
Range (min–max)	18–55	19–48	18–55
Mean ± SD	28.24 ± 6.50	28.44 ± 6.58	28.04 ± 6.45
**Gender**			
Male	127	65	63
Female	147	72	74
**Mandibular Canal at the Locations of IMTM**			
Below the root(s)	171	83	88
Canal between roots	22	10	12
Buccal to canal	34	19	15
Lingual to canal	47	25	22
**Cortical Bone State of the Mandibular Canal in the Apical Region of IMTM**			
intact without contact	0	0	0
intact with contact	189	92	97
disrupted cortical bone	45	45	40
**Probing depth(mm) pre-operation**			
Distal of M2 (Mean ± SD)	2.23 ± 0.66	2.25 ± 0.66	2.35 ± 0.65
Buccal of M2 (Mean ± SD)	1.91 ± 0.74	1.93 ± 0.75	1.89 ± 0.73

*NM* new method, *TM* traditional method

The surgery duration was shorter in the NM group than in the TM group (17.76 ± 3.78 min *vs.* 20.77 ± 3.48 min *P* < 0.05). Nonetheless, there was no statistical difference between the NM group and the TM group as to the post-operative pain (*P* > 0.05) (Table 2).

**Table 2:** Comparison of the duration of extraction and postoperative VAS score ($\overline{x}$ ± s)

	NM group(*n* = 137)	TM group(*n* = 137)	t	*P*
Duration of extraction(min)	17.76 ± 3.78	20.77 ± 3.48	6.85	< 0.001
Postoperative VAS score at 24 h(score)	3.60 ± 1.00	3.63 ± 1.23	0.22	0.83
Postoperative VAS score at 48 h(score)	2.70 ± 0.99	2.85 ± 1.06	1.18	0.24

*NM* new method, *TM* traditional method

With regards to the postoperative complications, there was no statistical difference noted in the incidence rates of postoperative hemorrhage, trismus, infection, and alveolar osteitis between the NM group and the TM group (*P* > 0.05). The incidence rate of lower lip paresthesia in the NM group was significantly lower than that in the TM group (1.46% *vs.* 7.30%, *P* < 0.05) (Table 3).

**Table 3:** Comparison of the incidence of postoperative complication [n(%)]

	NM group(*n* = 137)	TM group(*n* = 137)	*P*
Postoperative hemorrhage	2 (1.46)	4 (2.92)	0.68^a^
Trismus	12 (8.76)	18 (13.14)	0.25^b^
Wound infection	6 (4.38)	11 (8.03)	0.21^b^
Alveolar osteitis	1 (0.73)	2 (1.46)	1.00^a^
Inferior lip numbness	2 (1.46)	10 (7.30)	0.04^a^

*NM* new method, *TM* traditional method

^a^Fisher’s exact test

^b^Chi-square test

Significant difference was observed in the periodontal status of the M2 between both groups. The mobility of M2 at 30 days or 3 months post-operation in the NM group was significantly lower than that in the TM group (*P* < 0.05) (Table 4). The distal (2.38 ± 0.72 mm vs. 4.07 ± 1.47 mm, *P* < 0.05) and buccal (2.18 ± 0.60 mm vs. 3.72 ± 1.08 mm, *P* < 0.05) probing depth of the M2 at 3 months post-operation in the NM group were lower than those in the TM group (Table 5). The exposed root length of M2 at 3 months post-operation in the NM group was also lower than that in the TM group, with statistical significance (Table 6, *P* < 0.05). The clinical outcomes of the strategy in NM group was shown in Table 7.

**Table 4:** Comparison of the mobility degree of M2 post-operation [n(%)]

	30 days post-operation				3 months post-operation			
	NM group(*n* = 137)	TM group(*n* = 137)	Z	*P*	NM group(*n* = 137)	TM group(*n* = 137)	Z	*P*
**0 degree**	136(99.27)	122 (89.05)	3.61	< 0.001	137(100.00)	125 (91.24)	3.54	< 0.001
**I degree**	1(0.73)	10(7.30)			0	9(6.57)		
**II degree**	0	4(2.92)			0	2(1.46)		
**III degree**	0	1(0.73)			0	1(0.73)		

*NM* new method, *TM* traditional method

**Table 5:** Comparison of the distal and buccal probing depth of the M2 at 3 months post-operation ($\overline{x}$ ± s)

	NM group(*n* = 137)	TM group(*n* = 137)	t	*P*
Distal probing depth of M2(mm)	2.38 ± 0.72	4.07 ± 1.47	12.05	< 0.001
Buccal probing depth of M2(mm)	2.18 ± 0.60	3.72 ± 1.08	14.58	< 0.001

*NM* new method, *TM* traditional method

**Table 6:** Comparison of the root exposed length of M2 at 3 months post-operation[n(%)]

	NM group(*n* = 137)	TM group(*n* = 137)	Z	*P*
0 mm	136(99.27)	115 (83.94)	3.54	< 0.001^a^
1 mm	1(0.73)	16 (11.68)		
2 mm	0	5 (3.65)		
≥ 3 mm	0	1 (0.73)		

*NM* new method, *TM* traditional method

^a^Fisher’s exact test

**Table 7:** The effect of the strategy in NM group

	One Root	More than one root
Root luxation(s) – Crown	67	34
One root luxation – Crown – Root(s)	0	11
Partial Root(s) luxation – Crown – Root(s)	12	12
Root(s) subluxation – Crown – Root(s)	1	0

## Discussion

IMTMs in class C and horizontal position are “silent killer”. Due to the deep impaction, the prevalence of pericoronitis or other space infection is relatively lower than other types of impaction, thus it can be hardly noticeable to the patients. Nevertheless, deeply impacted IMTMs may give rise to root resorption, periodontal disease, distal alveolar bone resorption, and other concomitant symptoms of M2s [3, 4, 7]. The treatment of such deeply impacted IMTMs remains controversial. Some hold the view of conservative treatment as the procedure can be technically difficult, and the incidence of post-operative complications is relatively high using traditional method [3]. In this study, we aimed to introduce this new strategy to reduce the prevalence of the two main complications: inferior alveolar nerve injury and periodontal problems of M2, while increasing the surgical efficiency. Based on the current study, this novel method was shown to be more effective with controllable risks.

As the crowns of IMTMs in class C and horizontal position are mostly in close proximity to M2s, the highest resistance for removal of such IMTMs arises from the crowns of M2s [19]. The removal of the crowns of IMTMs during the operation may cause undesirable pressure on the M2s, or result in iatrogenic injuries to the roots, the crowns, or the restorations, which may adversely affect the pulp vitality of the M2s. Traditionally, the crown of the IMTMs was believed to be a priority to be removed, followed by the distal and buccal alveolar bone of M2s, especially the bone of the alveolar ridge. However, such maneuver is likely to cause postoperative periodontal complications to the M2s, such as root exposure, deep periodontal pocketof the tooth [16, 20]. In this “root removal first” strategy, the crown was luxated distally, which avoided the risk of iatrogenic trauma of M2s during operation and thus reducing the risks of nonvital pulp of M2s, especially those presented with pre-existing root resorption. Moreover, only a micro bone window was made on the buccal side of the IMTM’s crown and the alveolar crest of M2s can be preserved, which is beneficial to the healing and regeneration of alveolar bone, and the reattachment of attached gingiva. This was demonstrated in our study, thatthe periodontal health status of M2s in the NM group was significantly better than that in the TM group.

It has been reported in the literatures that transient inferior alveolar nerve injury occurs in 1–5% of all cases of surgical removal IMTM, and 0.1–0.9% of them sustained permanent inferior alveolar nerve injury [21]. For IMTM in class C position in which the root is usually adjacent to the mandibular canal, the incidence of inferior alveolar nerve injury after surgical removal can be as high as 11.8 to 19% [22, 23]. Similar to the previous study, the roots appeared to be attached with the mandibular canal, which was seen in the CBCT in this study, thus, the resistance for root removal during surgical removal of IMTMs can be considerably large [24]. Using the traditional method, the surgeon has to exert repeated forces to luxate the root, which often caused iatrogenic injuries to the inferior alveolar nerve. By contrast, in our new method group, the surgeon can easily removed the root as a result of the reduced root resistance.

Other treatment alternatives, such as coronectomy has been proposed for IMTM with a close relationship with the mandibular canal, to avoid possible damage to the inferior alveolar nerve caused by extraction. Coronectomy is indicated for IMTM without obvious inflammation around the root, the crown and root can be segmented at or below the enamel-cementum junction, and the cross-section is lower than the alveolar crest. The crown can be removed while the root is retained in the mandible [25]. Some patients may need a second operation to remove the root because of pain or inflammation caused by the remaining pulpal tissues in the roots, or the migration of the root to the alveolar crest [26, 27]. Removal via orthodontic traction is a procedure in which the bony resistance around the crown of IMTM is removed surgically to below the maximum circumference of the crown, and the force applied in occlusal direction using an orthodontic appliance. As the IMTM is moving away from the mandibular canal progressively, the tooth can be removed in a secondary operation. This method has a lower risk of damaging the inferior alveolar nerve, but the treatment period is relatively longer, the procedure is more complicated, and the cost is higher [28]. Prophylactic pericoronal osteotomy is indicated for impacted teeth due to minimal resistance of bone or adjacent tooth. The direction of IMTM eruption can be altered by surgical removal of a small amount of alveolar bone in the occlusal, distal, or buccal direction. The IMTM will be removed when the root is far away from the mandibular canal. This method has narrow indications because it is only suitable for young impacted teeth whose root has not yet completely developed. Moreover, this method is still in the exploration stage, and there is no standard surgical protocol published yet. [29]. By contrast, the “root removal first” strategy is safe and relatively low-cost method, which allowed effective surgical removal of deeply IMTM with a close relationship between the root and inferior alveolar nerve. It can not only shorten the duration of surgery but also reduce the risk of inferior alveolar nerve injury. However, more researches are required to further investigate the advantages and disadvantages of different methods. There were some limitations encountered in this study, the study involved a single chief surgeon and this is a one center-RCT.

## Conclusion

In conclusion, the “Root removal first” strategy is highly efficient, and reliable to reduce the incidence of inferior alveolar nerve injury and periodontal problems of M2, while reducing the total operating time.

## Data Availability

All data generated or analysed during this study are included in this published article.
